# Treating, Preventing, Feigning, Concealing: Sickness, Agency and the Medical Culture of the British Naval Seaman at the End of the Long Eighteenth Century

**DOI:** 10.1093/shm/hkab108

**Published:** 2021-12-15

**Authors:** Sara Caputo

**Keywords:** Royal Navy, patient’s history, seamen, naval surgeons, malingering

## Abstract

Seen as a crucial historical step in the development of ‘modern’ institutional healthcare, eighteenth-century British naval medicine has traditionally been studied from the point of view of the state and of physicians and surgeons: naval sailors’ attitudes towards health, medicine and their own bodies remain virtually unexplored. Using official and personal sources, this article sketches a ‘patient’s history’ of late-eighteenth- and early-nineteenth-century British ratings. Aiming to counterbalance Foucauldian interpretations, it highlights some of the ways in which individuals, even when apparently most powerless, confined in ships far from home, and controlled by rigidly disciplined institutions, could take responsibility for their health, successfully or otherwise, within, against or alongside the system. If the unprecedented administrative requirements of the French Wars strengthened and standardised top-down medical authority, they also brought opportunities for evasion and negotiation. This complicates established narratives of the relationship between modern medicine, the armed forces and power.

On 1 June 1813, the British frigate HMS *Shannon* fought a violent action against the American USS *Chesapeake*.^1^ In his journal, the ship surgeon reports the many wounded, including Patrick Ferreter, a 22-year-old ‘landman’ (inexperienced crewmember). Ferreter had been hit by the recoil of a gun, luxating his left humerus, but ‘he did not make it known until long afterwards’, at which point, ‘from its having been so long displaced’, it proved impossible to reduce the trauma. Even his discharge to hospital in Halifax ‘did not alter the case for the better’.^2^

When questioned about his behaviour, the young seaman

assigned as a reason for not applying at the time of the injury, that he did not wish to be on the sick list, whilst he was able to do any thing, at a time we were so short of hands, as after an action, and having another frigate in charge.^3^

‘Add to this’, the surgeon continued,

that the man could not make himself understood readily being able to speak only his own native Irish and [was] not at all acquainted with the customs of the service, having served in it only two days prior to the action.^4^

The story of Patrick Ferreter offers us a glimpse into a topic that is largely absent from the historiography on the Georgian Navy: a seaman’s attitude towards injury, medicine and his own body.

In Britain and Europe, the ‘long eighteenth century’ appears to mark a watershed in the relative position of patients and doctors. N. D. Jewson has famously argued that the rise of hospitalised medical care turned the ‘sick-man’, a significant, independent unit, into a ‘passive’ target of doctors’ practice, etymologically a ‘patient’.^5^ Mary Fissell has further highlighted the class struggle surrounding the right to ‘interpretation’ and management of bodies, with the poor being specifically and increasingly denied authority over their own health.^6^ By the end of the century, models of social and medical order and control had shifted from ‘small-scale’ ‘paternalism’ to impersonal large-scale management, discipline and even ‘compulsion’.^7^ A substantial scholarly literature has closely tied these changes to military and naval medicine, which were the first to develop, around this period, ‘authoritarian’ enforcement, systematic records and impersonal and ‘standardised’ treatments.^8^ This was paralleled by a more general disciplinary and rationalising turn in the administration and control of the Navy in particular—peaking with the unworkably draconian administration of Admiral St Vincent, and the attempted introduction of ‘panopticism’ in the dockyards in the early nineteenth century.^9^ Modern military environments, because of their institutionalised structures, seemingly provide a perfect case study for Michel Foucault’s model of medicalised control, of medical practice as yet another channel through which power can be imposed on the bodies of individuals.^10^ Thus, the prevalent idea among historians is that in the Navy ‘self-management was effectively denied the seaman’.^11^

Compounding this position are the enduring views of eighteenth-century observers, who portrayed seamen as ‘imprudent’, carefree and reckless—an extreme example of the lower classes’ need for direction.^12^ In this way, the combination of a supposedly very strict regime and supposedly irresponsible individuals has led to the relegation of the seaman to a marginal and almost incidental role in the medical history of the period. A growing body of research has illustrated the strategies of resistance, protest and dissent adopted by naval sailors by the time of the French Wars (1793–1815), and the ways in which they opposed or bypassed institutional constraints.^13^ This scholarship, however, has not yet reached the field of the history of medicine, which is, I argue, a pivotal one—particularly for switching the focus from collective to individual actions.

So far, naval medical historians have mainly debated the extent to which the new military medical regimes brought positive or negative scientific and therapeutic contributions. While James Watt highlights the benefits of controlled experiments, systematic record-keeping and ‘medical arithmetick’, and Patricia Crimmin those of improved prevention, others, like Geoffrey Hudson, qualify the notion of military medicine as ‘a good thing’, and J. D. Alsop criticises its instrumentality and potential ‘narrowness’, due to the exclusive focus on white male servicemen.^14^ What remains almost entirely neglected, however, is Roy Porter’s ‘view from below’: the history not of medical personnel and institutional provision, but of the specific beliefs of the sick, the methods that they adopted to keep and recover health, and their reactions to sickness more generally.^15^

The extent to which the early-modern sick person truly was a subordinated entity, a passive ‘patient’ deferring to ‘closed’, ‘esoteric’ medical knowledge and authority, has long been questioned.^16^ For the eighteenth century, the Georgian ‘medical marketplace’ of treatments and practitioners, from which the sick could choose, is now a recognised historiographical concept.^17^ Even extremely ‘disempowered’ groups like enslaved people in American plantations, it has been shown, maintained a complex structure of beliefs and medical self-help practices.^18^ The enslaved differed from seamen, nonetheless, in that their pursuit of such treatments was due to the inadequacy and weakness of official medical provision: the naval healthcare system was at times inadequate, but by the end of the century we tend to imagine it as the opposite of ‘weak’. This article will question the notion of a linear doctor–patient hierarchy even in a context supposedly ushering Jewson’s post-‘medical marketplace’ era, and where doctors’ authority and control were in theory rigidly defined.

During the Revolutionary (1793–1802) and Napoleonic Wars (1803–15), the Navy recruited an unprecedented number of men—by 1809 over 140,000 were in active service, nearly 70 per cent more than in 1760.^19^ In the Seven Years’ War (1756–63) the fleet had operated mainly across five foreign stations (East Indies, Jamaica, Leeward Islands, North America and the Mediterranean), but by the end of the Napoleonic War these had become 10, besides the Channel and Ireland.^20^ Warships also grew by over one-third in average size, resulting in larger shipboard communities.^21^ If the new scale of administration led to attempts to create stricter and more efficient management practices, it also meant that the seamen became harder to evaluate and control as individuals, and their range of experiences and backgrounds broadened. This, I will argue, often left naval ‘hands’ some scope for exerting medical control over their bodies, and perhaps offered them increased leverage and competence to do so.

The concept of ‘agency’ has been criticised by some scholars because of the main senses in which it is usually deployed: as a synonym on the one hand of ‘humanity’, which leads the historian to state the obvious, and possibly validate unsavoury socio-political doctrines (if it is necessary to argue that enslaved persons, workers, sailors had the capacity to act independently, as humans, the counter-argument that they did not is implicitly accepted as worth considering), and on the other of ‘rebellion’, which is reductive.^22^ This article, however, taking for granted that naval sailors were humans, with free will and intellect, will aim first of all to counterbalance a literature that has most often emphasised the constraints in their situation, rather than the opportunities, and second to show the specific ways in which this ‘humanity’ could express itself. Most importantly, not all the forms of ‘agency’ analysed here could be construed as rebellion: the medical needs and interests of the seamen and those of the state that they served were often divergent, but equally often could they come to coincide, however temporarily; what is of interest here is recovering how the *methods* chosen to reach certain goals could differ, and what say the individual could have on such methods. Naval sailors’ medical culture did not necessarily entail evading the surgeons and spurning their expertise, unless further motives intervened; but interactions with the Navy’s medical personnel were negotiated, dosed, calculated and supplemented by other options and beliefs, often specific to the wartime context. The delicate power compromises that, according to N. A. M. Rodger, operated in the mid-century fleet instead of discipline still survived in medical matters by the turn of the nineteenth century.^23^ They simply found ways to operate alongside, and through, the very language of discipline, exploiting the Navy’s at times desperate need for ‘manpower’.

A final caveat is needed. All the historian can do is to observe these men’s behaviour, whether it fitted within the rules of the service, and sometimes whom it benefited. Beyond that, motivations are difficult to assess in their true depth. Numbness to death, self-harm, ‘concealment’, and often malingering, all treated in what follows, are actions to which no superficial explanation could ever do justice. The question of mental health in the Navy is a complex and important one, which is currently receiving long overdue attention.^24^ Remaining aware of the constraints that the mind quietly imposes on individuals should be a perpetual deterrent for any account proposing naively rosy views of human freedom. This article is not meant as one such account. Life in the eighteenth-century Navy was harsh, unforgiving, and coerced in ways that we can perhaps not even imagine; many of the actions discussed in what follows stemmed from sheer desperation. However, it is necessary to show that the Navy as an institution was not all-powerful in its control of individuals’ health and bodies. It always had to compete with other forces, including the seamen’s own views and actions. Institutionalisation itself engendered reactions, loopholes and opportunities.

Overall, there are at least three reasons why naval sailors of the French Wars offer an important example of medical autonomy vis à vis the Foucauldian state: their professional and military value, their unusually broad range of experiences, and finally, it can be suggested, a certain peculiar mindset, or at least attitude, that came with a dangerous and often forced job.

## The Navy’s Medical Provision

The quality of life in the eighteenth-century British Navy has been a matter of lively debate among historians. Traditional narratives highlight miseries and suffering, ruthless impressment (forced recruitment) practices, harsh punishments and general neglect of the individual’s welfare.^25^ Others, however, have argued that often Royal Navy seamen were volunteers: for many of them, serving was a rational choice, with the comparatively low pay counterbalanced by food, pensions and free medical care probably better than what they could have afforded ashore or aboard merchantmen.^26^ Naval surgeons suffered a bad reputation, undoubtedly justified, at times, since the Navy absorbed many unsuccessful practitioners, ‘young men who could not procure any other employment’.^27^ Nevertheless, ships’ surgeons, their chests and their journals had to pass formal examinations.^28^ Surviving logs, moreover, show detailed day-to-day attention to each patient, sometimes treated with sizeable amounts of medicines.^29^ Comparing naval and civilian practice on the basis of success statistics, as James Watt does, is made problematic by the limited samples and huge environmental variables.^30^ Systematic prosopographical work, however, has shown that in the French Wars the average level of education and competence of naval surgeons was very high, as illustrated by the rates of professional achievement when they re-settled into civilian life after 1815.^31^ Overall, Erica Charters has convincingly argued that the eighteenth-century fiscal-military state had important political, practical and ideological reasons for maintaining sustained ‘attention to the welfare of its armed forces’.^32^

Therefore, whilst life at sea itself caused medical dangers, in some cases a seaman, by enlisting in the Navy, could in fact *widen* the portion of the medical marketplace at his disposal. Dr Thomas Trotter, ex-Navy surgeon and Physician to the Channel Fleet in 1794–95, wrote that he had ‘frequently known seamen enter on board a man of war, for no other reason but the cure of the Venereal Disease, which they were not able to pay for, on shore’.^33^ We shall see, as a recurring theme throughout this article, that sailors regularly sought the naval surgeons’ help, and this was a form of agency in itself. Of course, once in the service, their autonomy of choice was somewhat curtailed.

Most problematic, to our eyes, is the frequency of medical experimentation on eighteenth-century servicemen, facilitated by the fact that they were ‘coerced, plentiful, and the intended beneficiaries of any improvements’.^34^ The medical and scientific use of the bodies of disempowered individuals, before and after death, is a typical feature of a Foucauldian biopower system, targeting ‘voiceless’ groups.^35^ British naval seamen subjected to these trials were not necessarily consulted, and sometimes lost their lives, as in the tests against dysentery conducted at Haslar Hospital in 1795.^36^ However, in the eighteenth century, ‘medical trials’ were by no means confined to servicemen; civilian doctors conducted them not only on the poor in charitable hospitals, but also on their elite ‘private’ patients, and even on their own children, and on themselves.^37^ After the twentieth century, we feel strongly about norms and guidelines on ‘human experimentation’, but these need to be historicised, particularly as relates to the availability of safe treatment options.^38^ Eighteenth-century military surgeons were often averse to any experiments that deliberately caused new ‘danger’ to the subjects, as opposed to holding potential benefits.^39^ When death is the alternative, a dubious or experimental cure can be more welcome to the sick than no cure at all. During some trials of nitrous acid fumigations aboard the *Union* hospital ship, in 1795, the surgeons personally stood in the ward with the patients, who, they claimed, developed great ‘faith’ in the procedure, and even assisted in performing it.^40^

In general, the Admiralty dedicated growing attention and substantial resources to seamen’s health, mainly because the British state needed skilled seafarers, and faced an increasing shortage.^41^ The notion of ‘utility’ as a qualifying criterion for medical treatment is precisely one of the pillars of Foucault’s argument, and it can be read as a dehumanising form of objectification.^42^ Seldom considered, however, is the power that it also gives to the sick. If sailors, and especially the highest-rated ‘able seamen’, truly were such a valuable demographic category, we might suspect that trials carried out on them would tend to be *less* reckless and unjustified than those on other groups, at least at the level of institutional strategy. As shown by Charters, even in the middle of the eighteenth century, when recruitment pressures were lower, the Admiralty Sick and Hurt Board were indeed very cautious in selecting which cures to authorise for trial, and in proceeding gradually, starting with small groups, and interrupting the tests if necessary.^43^ A relatively well-developed medical provision and carefulness with untested treatments signify that the sailor was held as valuable by the institutions he served. This was, as will be seen, an important precondition for negotiating power.

## Treating and Preventing

If seamen’s value to the state ensured that they were offered some medical treatment, this does not automatically mean that the standardised care provided would be sufficient, appropriate or congenial to the individual. The sailors then acted to address this discrepancy, often using special knowledge and tools.

Long confinement at sea obviously limits individuals’ options: a primary issue are the environmental conditions from which disease springs, which were the object of much attention among eighteenth-century medical practitioners.^44^ In 1758, for example, the surgeon Thomas Reynolds opened his recommendations to Lord Anson regarding the health of seamen explicitly stating that he did ‘not intend to present any thing under the denomination of medicine’, but rather deal with preventive ‘oeconomy of the Sick’ (i.e. matters of clothing, victualling, and lodging).^45^ The difference in living conditions between officers and common ratings was striking, and contemporaries noticed its impact on health: better provisions and separate quarters made officers less vulnerable to scurvy and epidemics.^46^ The sources are replete with stories of ‘gallant, generous, and beneficent’ superiors who out of ‘compassion’ shared their provisions with the lower deck, even beyond their means, gaining immense ‘gratitude’ in return.^47^ However, this was not simply seen as a matter of free charitable gifts: care on the part of officers was clearly cast as a duty, and this stemmed not only from the material constraints of shipboard conditions, but also from the perceived incapacity of lower-deck sailors to take care of themselves. Over the following decades, this perception survived, almost unchanged, the slow transition from paternalistic to state-disciplined models of naval healthcare.^48^

In his 1795 text on naval medicine, Gilbert Blane, ex-Physician to the Fleet, repeatedly emphasised the importance of ‘discipline’ and good ‘management’ for shipboard health, laying the responsibility squarely on the officers’ shoulders, and thus questioning the ‘unthinking’ ratings’ discernment.^49^ Showing national pride, contemporaries praised the British fleet, by comparison with those of other countries, because of the way in which it took good care of its men and retained efficiency through discipline: ‘all seamen, if not naturally dirty, are extremely careless, and therefore require looking after more than most other men’, observed a British naval surgeon deployed on a Russian ship in 1814; the Russian officers’ complete neglect to check whether the seamen shaved, changed, washed, or where and how they slept, together with the incompetence of surgeons, was for him the cause of the high incidence of sickness in that Navy.^50^ The view apparently prevailing in the Russian fleet, that ‘the health of the men was their affair alone and no other person’s’, was intrinsically unacceptable in the British service.^51^ The depiction of sailors as ‘childlike’ and ‘improvident’, as mentioned above, was overall commonplace in this period, and part of the rhetoric of control.^52^

However, sparse mentions in Reynolds’s very text reveal a broader picture, with seamen taking the initiative: moving their hammocks up on deck when disease was raging (‘from prudence, or any other motive’, he grudgingly admits), or, if they had the skill, re-sewing the poor clothes issued to them into garments more suitable to the climate.^53^ Intuition and experience could apparently suggest to some sailors the same solutions for which gentlemen and doctors argued at length in print. Men like the cook’s mate who ‘complained of the offensive smell of the sheets’ at Haslar, in 1797, and managed to have them changed (though not before catching a lethal smallpox infection), did not hesitate to make their voice heard.^54^ When living conditions were excessively unhealthy, seamen were even prepared to rebel, and, unlike land people, sometimes had the instruments to do so. The great naval mutinies of 1797 had complex causes, but among the requests were ‘a sufficient quantity of vegetables’, better care for the sick, and prevention of the embezzlement of medicines.^55^ Even in a heavily ‘policed’ place like Greenwich Hospital, protests and resistance of various kinds were frequent among pensioners.^56^

We should be wary, anyway, of seeing seamen’s medical interventions only when they took the shape of rebellion. Many sailors trusted their surgeons and actively collaborated with them in formulating a diagnosis, and even the treatment. In December 1807, Thomas Crew, the surgeon of HM Brig *Contest*, treated 23-year-old Ellis Eslam for a very bad cough, pains and expectoration, noting that the seaman had ‘been always Subject to a difficulty in Breathing, particularly in cold weather’ or when exercising heavily. ‘I am given to understand the Source of His disease is Hereditary’, he initially wrote. A few days later, however, a detailed patient anamnesis changed his thinking:

I find from very minute inquiry that this poor man attributes the origin of His complaint to working at the trade to which He was brought up (namely the needle making) from which time (as well as while employed) He always laboured under ill Health, and through medical advice went to Sea for recovery. I was (at first) at a loss to find how his ailment could have made so great a Progress in so short a time as p[e]r journal. He further states the bad Effects he always found to result from Colds.^57^

Phthisis, ‘grinders’ consumption’ (asthma) and early mortality were indeed common among needle-makers, due to the inhalation of stone and steel dust.^58^ Eslam had a very good notion of what his medical issues were, what improved them and what worsened them, and the surgeon found it useful to listen to him. This was not an isolated case. In HMS *London*, in the summer of 1800, the seaman John Schull was sick with catarrhal fever: ‘Being accustomed to be bled in Hanover and different parts of Prussia for every disease he had in that Country: requested that he again might be bled’. ‘Accordingly’, the surgeon concluded, ‘ten ounces\blood/was taken from his Arm’. A side note in the journal further clarifies: ‘It is a common request among the Foreigners on board here, especially Germans, Prussians and Maltize to be bled for every disorder’.^59^ This is a good illustration of how seamen from all walks of life and corners of the world came aboard with their specific medical beliefs, and negotiated their cure with the surgeons, who—contrary to what the public rhetoric might suggest—sometimes complied with their wishes. Some naval patients were even able to refuse treatment, as shown by the examples of those who successfully resisted amputation and smallpox inoculation.^60^ Even when the sailor and the surgeon did not openly clash, their relationship was much less unidirectional than we might assume. As Patricia Crimmin has argued, ‘local regimes’, rather than centralisation, remain the best explanatory model for naval administrative dynamics, into the late eighteenth century.^61^ Ships (and fleets) were distinct, far-travelling communities, under specific officers, and need to be studied as such.

What about choice of treatments beyond interaction with naval surgeons? Ashore, we saw, people could draw on a vast and diverse ‘marketplace’ for all purses, including not only university-educated practitioners, but also home remedies, neighbourly help, ‘alternative’ medicine, magic, and what the medical establishment classed as ‘quacks’.^62^ Physical confinement aboard ships and eradication from land communities could, one may assume, constrain naval seamen’s access to these opportunities. In reality, however, especially as the Navy scaled up recruitment and foreign recruitment, sailors also formed their own new, cosmopolitan and multi-ethnic shipboard communities.^63^ Moreover, in 21, almost uninterrupted years of global conflict, they also came into contact with local people in ports not only in Britain but all over the world, through bumboats even when they were not granted shore leave. This, it can be argued, despite making them potential, inviting targets for fraud,^64^ in some ways actually broadened the marketplace at their disposal.

Dr William Turnbull, in condemning the former practice of fining seamen for venereal diseases (a sizeable 15s, until 1795), remarked that this led them to ‘put themselves into improper hands’, rather than relying on their surgeon.^65^ Indeed, whether aboard or ashore, sailors had access to some of the vast medical marketplace that catered to this type of complaint—as Dr Trotter put it, a ‘herd of quacks and itinerant practitioners who frequented the sea-ports, and preyed on the credulity of our men’.^66^ Early in 1802, for example, HMS *Princess Royal* had a spate of seamen who openly told the surgeon that they had suffered from venereal ulcers and cured them autonomously (one man ‘by the application of lunar caustic [silver nitrate] without any medicine internally’), or had ‘been under the direction of some person on shore’ in Portsmouth, ‘taking some mercurial preparation’.^67^ If they wanted the men to come to them, naval doctors had to foster an atmosphere of trust and support rather than punishment—because their care remained an option among many. This mirrored the attitude adopted by the Navy when faced with seamen’s agency on other issues: for example, even if desertion was potentially a capital offence, it was rarely punished as such, and the crown periodically issued general pardons.^68^ If deserters were put in a position where they could never consider returning, their skill was forever lost to the fleet.

Sailors found remedies and preventatives for various conditions, more or less successfully. In October 1804, an ordinary seaman in the crew of the prison ship *Le Pegase*, suffering from bubonocele, had it ‘reduced by his comrades’ (which provoked a hernia humoralis).^69^ Dr Blane observed how seamen, when in port, exchanged their rations of salt for sugar, then mistakenly considered an antiscorbutic (the Sick and Hurt Board only recommended its systematic issue with lemon or lime juice in 1807).^70^ Trade in other products, often healthier than preserved rations, would likewise occur. Admiralty regulations forbade the sale of ‘Fruit or Strong Liquors’ aboard, for reasons of ‘Cleanliness’, but the rule was clearly ignored.^71^ A sailor whose ship spent a few days near Cadiz, in December 1795, noted that local boats came over to sell the men bread, ‘Garding Stuff’ (‘Cabbage, Onions, Turnips, Redishes &c.’) and fruit (‘Pomegrannuts, Oringes, Figs, Grapes, Raisons &c.’).^72^ The Portuguese in this place sold fruit ‘very cheap’, according to another seaman.^73^ Not only did this allow the men to vary their diet, but it also introduced them to new products that were virtually unknown in Britain, especially to persons of their social background: ‘Pomegrannuts (you may read of in Scriptur)’, the first sailor explained to his mother, ‘are the size of Oringes, much about the cullur of Mellow Apples, the Shell or Skin, much about the same thickness as that of Oringes, the inside something like that of Goose Buerreys and tastes much like a Bramble-Buerry (or Bummelcite [blackberry] as you call them)’.^74^

Similarly, the travelling itself, and the heterogeneous crews, could perhaps make seamen aware of different treatments and lifestyle regimes, even though this question requires further study. As shown by Wendy Churchill, non-European servicemen in particular were often allowed some ‘autonomy’ in their victualling, living and clothing choices; this stemmed, again, from a desire for military efficiency, coupled with contempt and racial beliefs that different ‘constitutions’ had different needs.^75^ Such allowances, however, do not fit well with a standardised model of medical discipline, and would have meant that many European seamen came into close contact with alternative (and, some doctors realised, in certain climates more efficacious) dietary and hygienic practices.^76^

## Feigning and Concealing: The Seaman and His Body

Life at sea, of course, remained extremely dangerous. Sailors’ constant exposure to death and sickness, however, had two consequences: first, it gave them an in-depth knowledge of what ‘illness’ looked like, what provoked it and what the naval establishment’s reactions to it were. This was very precious information for men whose goals had ceased to be aligned with those of the Navy—that is, for men who wished to exit the system. Second, and more tentatively, it could be argued that a precarious, high-stakes life, with abundant reminders of the fragility of human bodies, could raise seamen’s bar for what was tolerable suffering, making them more difficult to coerce into anything. These two aspects will be examined in turn.

The most simple way in which seamen could use medical conditions as their ticket to freedom was exploiting the difficulty for some hospital structures, especially those based on land, to contain desertions.^77^ This should not be exaggerated, particularly for later in the century: for example, if we take them at face value, the musters of Haslar Hospital only show four patients marked as ‘Run’ for the whole of 1798 (against 4,595 ordinary discharges), and one each for 1801 and 1813 (against 2,841 and 2,763 discharges, respectively).^78^ Plymouth Hospital also had a single escape in 1801.^79^ However, there would have been some variations depending on location: as shown by Guenter Risse, for instance, the registers of the Royal Infirmary of Edinburgh list under the category of ‘irregular’ (including both runaways and disciplinary expulsions) 13 sailors’ discharges (6.2 per cent) in 1791–94 and 24 (7.3 per cent) between 1795 and 1800.^80^ The naval authorities were clearly alert to the problem: the windows at Haslar were provided with ‘Iron Railings and Bars’, and the same was true of hospitals in naval bases abroad, for example in Menorca.^81^

Even if he could not escape, moreover, managing to be sent to a land hospital had multiple advantages for a sailor. As the Governor of Haslar complained in 1797, among the incoming patients

there are a vast number who avowedly come in to gain a point, which is, either through a long struggle and great Imposition to be put on the list for survey, with the hope of Invaliding, to hang on the Hospital as long as possible from the great comfort of their situation or endeavor at effecting their escape.^82^

As a solution, he suggested sending those whose ill intent was clear to the ‘Convalescent Ship’, ‘which they hate to go to on account of the confinement’: allowing them to stay at the hospital only made them ‘daily… more hardened’ and ‘set the ingenuity of others at work’, acting as inspiration.^83^ Overall, it appears that discipline in sick quarters could easily slip: officers surveying the state of the patients in North Yarmouth, in 1797, found that the seamen were ‘at times capricious in their Diet’, and instead of milk and broth ‘they generally insist on Bread & Cheese’ (which came with more beer), taking advantage of the fact that there was ‘no authority to enforce the former however necessary that Diet may be for them’.^84^ Once again, behind the simplified narrative of irresponsibility we can distinguish men who happily ignored medical regulations, even to their own detriment, having reached a place of rest and relative freedom.

The easiest route to a hospital was of course genuine illness. Another way to obtain this result, however, immediate respite from duty, or even official discharge from the service, was a sufficiently convincing performance of illness. Malingering has always been common in armed forces around the world, and a substantial historical and medical literature exists on this phenomenon during different conflicts.^85^ We should remember that malingering is not only socially and historically constructed, but also easy to confuse with factitious disorder, even in the presence of living patients.^86^ Therefore, it would be a gross generalisation always to assume a shrewd calculation on the sailors’ part. However, during the French Wars, systematic impressment, lack of shore leave and the recruitment of inexperienced ‘landmen’ on an unprecedented scale may have encouraged an endemic culture of seeking relief or discharge—through malingering, besides (as is well known) outright desertion.^87^ Seamen feigning complaints posed a complex challenge to the authority of surgeons.

Even today, mental health conditions are still mostly diagnosed on the basis of self-reports, which means that they lend themselves to simulation: post-traumatic stress disorder (PTSD) is estimated to be the most commonly feigned complaint in modern Western militaries.^88^ For the same reason, in the eighteenth-century Navy some doctors regarded mental illness as a popular choice among malingerers. However, naval surgeons also faced the additional challenge of the lack of imaging, microbiological or biochemical diagnostic tools: even in the case of many physical complaints, positively unmasking a simulator could be a formidable task.^89^ Although eighteenth-century medicine, as shown by Fissell, was beginning to discount patient ‘narratives’ in favour of ‘physical diagnosis’,^90^ this power shift had less traction in a context like the Navy, where patients were eager to be under medical care, and therefore deliberately selected those complaints which remained on the opaque frontiers of contemporary diagnostic medicine.

Thomas Carruthers, surgeon in HMS *Dreadnought* in 1803, compiled a short essay ‘Of Impostors’, attempting to explain and classify malingering behaviour. The men, he wrote, were ‘induced’ to it ‘from fear, bashfulness or from lucre’. As he explained, the problem was that a physician or surgeon ‘can only judge from the symptoms’, ‘their presence or absence’. Yet ‘artful people, by a specious tale, and by feigning disorders where much is to be known from their own confession, may cause a good deal of difficulty to discover the truth’. He then proceeded to suggest a method to attempt to counter this behaviour, through thorough examination of symptoms and appearance, interrogation of the patient and other witnesses with questions ‘so framed as to confound them’, or frequent sudden visits when the sick man ‘least expects it’. A proper power struggle of wiles thus raged between the sailors and their doctors: the latter used their position of authority and scientific expertise, but the former used their own lived and witnessed experience of sickness to refine their impostures. As Carruthers observed, ‘many diseases’ could ‘be feigned, particularly by a person who has before suffered from them’, and if they did not entail fever, but had diagnoses depending entirely on the patient’s word. However, he downplayed sailors’ ability, stating that only ‘a few’ were ‘generally’ used: ‘Epilepsy, melancholy, foolishness, possession by evil spirits, and fascinations’. Some methods suggested for uncovering pretended epilepsy were brutal, like pressing a hand on the man’s mouth and nose to prevent him from breathing, or applying burning coals to his hands during a supposed episode, and observing his reactions.^91^

Carruthers was a very alert and severe doctor: the butcher’s assistant on his ship, William Carters, complained of a stiff left arm from an old wound, requiring a sling, but showed no convincing symptoms, and ‘was observed making use of both hands’ at work. Carruthers had the man’s grog stopped, and him put ‘to swing Shot on the poop every day to shame him, and as an Example to others not to do the like’.^92^ Other doctors also employed harsh measures to break malingerers: the Governor of Haslar, for example, recommended that those who ‘protract their cure, by secret and improper applications’ ought to be sent to the convalescent ship, and ‘kept on low diet’, at the complete discretion of the medical personnel, ‘to destroy the hope of their being Invalided or Running’; those who faked rheumatisms, too, were to be transferred to that ship, or recommended for service in a warm climate.^93^ However, the surgeons did not always emerge victorious from this kind of struggle, for various reasons.

First of all, medical records only report the cases of men whose deception was discovered. Yet many more seamen may have succeeded in their goal, making their trick invisible to the historian. We know that a few sailors started off with a relatively high opinion of surgeons’ expertise, and therefore sceptical of their chances, but were pleasantly surprised. Just after the end of the American War, the American seaman Joshua Davis was eager to return home, but could not find a passage, so he had to join a Navy ship stationed in the Medway. Finally, he managed to obtain a spot in a merchant vessel bound to the United States, just before his ship was due to sail: he decided to pretend sickness to be discharged to hospital. He confided in a messmate, who believed that Davis ‘could not deceive the doctor’, but agreed to help him. Davis proceeded to ‘go to my hammock, drink a pint of vinegar a day, rub allum on my tongue to make it white, and rap my elbow against the ceiling to make the pulse beat’. Surely enough, the surgeon fell for his trick and sent him to hospital, where Davis secured a discharge by bribing a surgeon’s mate. (Unfortunately, he immediately went out frolicking in the fields with friends and was spotted by his former officers).^94^ Davis Bill, another American, was given ‘a small piece of lake red’ by a shipmate, in 1799: ‘with this’, he later wrote in his memoirs, ‘I laid a plan to try the skill of the surgeon, which succeeded even beyond my expectation’. He made himself sick with tobacco-juice, and used the red colour to make it look as if he was vomiting blood: the ‘good surgeon’ promptly dispatched him to Plymouth Hospital, where, he concludes, ‘I found a great number of invalids…; some appeared almost as hearty as myself’.^95^ American memorialists had good political motives to make British officers appear foolish, but these seem plausible glimpses of the seamen’s hidden manoeuvrings and shared arsenals of tricks—a collaborative culture of medical deception. The surgeons were not infallible.

Moreover, even when they suspected malingering, surgeons could have good reasons to look the other way. Sometimes, getting rid of a man who was clearly not eager to work and became a drain on the medics’ time benefited both parties. Private Marine James Robinson ‘had been a constant attendant in the Cocke [*sic*] pit [sick berth]’ of HMS *Captain* ‘from the day he came on board’, complaining of rheumatisms and a lame right arm, but he had failed to persuade Mr Farquhar, the surgeon, to put him on the sick list. Farquhar ‘had great reason to believe that this Man pretended to be much worse than he really was’; ‘by perseverance however he at last gained his point’, the surgeon eventually wrote in May 1798, ‘both his Officers and myself being glad to get clear of him by sending him to the Hospital’.^96^ This followed the same principle by which some naval commanders made sure that their worst people deserted.^97^ If generally doctors were more inclined to believe men of ‘good character’, however implausible their complaints,^98^ occasionally there was something to be said for ‘believing’ the most undisciplined, too.

This process, however, tested the very edge of shipboard officers’ and surgeons’ authority. John Rose, seaman in HMS *Lion* in 1813, complained of back pain from an accident which his petty officer denied had even taken place, showed no external signs of injury, claimed contrasting and varying symptoms, and changed position ‘when he thought himself unobserved’: his surgeon, John Todd, declared him an impostor and sent him back to duty, but the man ‘continued to make the same complaint, stooping & of no use on board’, turning into ‘a burthen to the ship’. Exasperated, the surgeon discharged him to hospital with a terrible reference and no smart ticket, ‘in order that I might comply with the frequent appeals he has made against my humanity, and that if really Ill he might derive benefit from your treatment, if skulking he might be placed under more rigorous discipline than I have in my power to enforce’.^99^

Todd was sure about Rose’s case; surgeons were even more disempowered when they were not, and had to concede a victory to the simulator out of caution, rather than simply annoyance. Thomas Simpson, for instance, surgeon of HMS *Arethusa* in 1806, reports a case of headache: the patient, a marine notorious aboard for imitating animals in exchange for grog, was

a sottish drunken fellow, of such a ghastly wretched appearance in general that it is a difficult matter to ascertain at anytime whether he is in health or otherwise especially if it is convenient for him to affect indisposition – which is very often the case.^100^

The man, Simpson assumed, was probably just feigning sickness to avoid being punished for drunkenness, but even a case like this was given the benefit of the doubt and put off-duty.^101^ Similarly, Thomas Hendry, surgeon of HMS *Ambuscade* in 1801, suspected that the marine Bernard Wels(h)ford was a simulator. The young man complained of heavy pain in the head and side, often ‘roared so loud as to prevent the Ship Company resting’, refused food, did not leave his hammock, and vomited after taking tea or gruel. The vomit, according to Hendry, was possibly self-induced, and there were no other symptoms of illness. Yet he still sent the man to Plymouth Hospital, albeit with a bad character reference: ‘Altho’ I had every reason to suppose this Man feigning Sickness’, he wrote, ‘still I was convinced that serious complaint may exist, without any marked dangerous symptoms’.^102^ Welshford managed to remain in hospital until 12 March, with a generic diagnosis of ‘Debility’, before being discharged back to ‘Quarters’, and thus presumably to duty.^103^

A telling comment was included by the surgeon of HMS *Overyssel*, Alexander Gordon, in his ‘General Remarks’ for the year 1797–98:

I have to regret that we have had a great deal of trouble & perplexity with feigned & artificial complaints, & that the present times compel surgeons to proceed with caution, as severity, in such cases, though proper, might be productive of unpleasant consequences.^104^

The surgeons undoubtedly feared medical mistakes, as was the case for Hendry, but a clear disciplinary concern is also evident in Gordon’s text: he worried about spreading discontent, given that his ship was stationed at the Nore, and the great mutinies had just been suppressed that year (‘the present times’). Harshness would not be tolerated too well by the men in the specific circumstances, and the implications of an error of judgement on his part—mistrust towards his medical provision—could be catastrophic. This situation was perhaps peculiar to the precarious disciplinary balance of the fleet in the 1790s, a decade in which draconian repression had to be weighed against the threat of revolutionary ideals, and social negotiation acquired a new, sharper edge.^105^ Some surgeons and surgeons’ mates were sent ashore by their crews during the 1797 disturbance, and had to be replaced by new appointments; their fate was left suspended for almost a month, until eventually the Admiralty allowed the Sick and Hurt Board to grant them new warrants, but suggesting that they be assigned to ships which were not in the same ports as the ones from which they had been ejected.^106^ Even if in theory they had immense power over their crews, then, in practice surgeons operated under heavy constraints. Especially given the diagnostic limitations of the time, the surgeon’s conscience, reputation, and job itself could be on the line every time he decided to reject his patient’s word—even if that patient was the lowliest of sailors, or marines.^107^

In addition to this, naval surgeons often had to deal with patients who could be ruthless towards their own bodies, thus undermining the very essence of a doctor’s authority.

A leitmotiv in seamen’s memoirs from the time, deeply entangled with social precepts of performative masculinity, is an acquired indifference or numbness towards danger, injury and death.^108^ In his *Recollections*, Commander Anthony Gardner describes how, during an action he fought aboard HMS *Panther* in the American War, a man was ‘cut in two by a double-headed shot… and the lining of his stomach (about the size of a pancake) stuck on the side of the launch’. The ship butcher allegedly ‘began to scrape it off with his nails, saying, “Who the devil would have thought the fellow’s paunch would have stuck so? I’m damned if I don’t think it’s glued on!”’.^109^ During his first days in the service, in the early 1820s, a 17-year-old was absolutely ‘shocked’ at the general ‘carelessness’ with which his new shipmates treated the sudden death of a man: ‘I verily believe that a dog, if it had died in the same way, would have caused as great a sensation in the ship as the death of poor Jack’.^110^ Of course, desensitisation to others’ suffering does not imply equal indifference to one’s own, whatever the impression of personal bravery that the men tried to cultivate. However, such a pervasive presence of death and terrible injuries would have likely made less serious alternatives look relatively minor, and overall a convenient sacrifice.

In the constant struggle against surgeons seen above, which required high levels of plausibility, seamen’s malingering easily blurred into self-inflicted injury. Again, ‘disease forgery’ (simulation that goes to the extent of actually manufacturing symptoms, or even self-harm), is a common occurrence in militaries.^111^ The context, however, dictates how far individuals are prepared to go with this, and some in the late eighteenth-century Navy went considerably far. The debate on the brutality of impressment is still open among naval historians,^112^ but there is little doubt that non-volunteers served on a larger scale than ever before, and—due to the sheer duration of the conflict—deployments away from home could last far longer. Even within the highly idealised picture that he painted of the ‘British seaman’, Dr Trotter observed that some impressed and disaffected men ‘assume diseases, to be an object for invaliding’.^113^ The sailor

employs caustics to produce ulcers; inflates the urethra, to give the scrotum the appearance of hernia; and drinks a decoction of tobacco, to bring on emaciation, sickness at stomach, and quick pulse.^114^

In a text praising the brave British Tars, Trotter dismissed these as rare, temporary occurrences, patiently dealt with by the paternal surgeons, and not resulting in any punishment. The story that we distinguish through his lines, however, is potentially very different. In his 1843 treatise on malingering, Hector Gavin presents a 366-page-long list of tricks and deceptions used by soldiers and sailors, often during the French Wars, to pretend illness; many of these, such as introducing foreign objects into orifices, or smearing the gums with corrosive substances to simulate the symptoms of scurvy, frequently caused real damage, if not the very complaint that had been feigned.^115^ Beyond a supposedly superficial and temporary stimulation of symptoms, examples of men deeming deliberate permanent disabling a worthwhile sacrifice are easily found in naval records.

In 1807, the Black Spanish prisoner of war Peter Salagara, with family at home in Cuba, ‘cut off three Fingers from his left Hand to prevent his being able to serve in His Majesty’s Navy’.^116^ In November 1811, Private John Wheeler of the Royal Marines was condemned to suffer 200 lashes and forfeit all his arrears of pay, for having ‘attempted to disable himself’ by cutting the tendons of his left wrist.^117^ Although he firmly maintained that it had been an accident, this went against both his messmates’ testimonies and unquestionable medical evidence, and the court decided to make an example of him. The list could go on.

Seamen could also harm themselves less directly, by concealing their illness. This behaviour went against the expectations of the naval medical establishment that seriously injured or sick men come forward, to be disposed of or cured and made fully effective again (and to prevent contagion or other danger to the rest of the crew). Concealment was in many ways opposite to malingering, but often it too aimed for specific personal gain, such as being able to enter or remain in the service, or avoiding punishment. The Navy had a serious ongoing problem with the quality of the men recruited at its rendezvous, with many being later found ‘improper’ or outright ‘unserviceable’ upon closer survey.^118^ Concealing chronic and debilitating illnesses at the enlistment stage, in order to receive the ‘recruitment bonus’ and be discharged shortly after, is a trick that servicemen have often adopted in wartime.^119^ The large-scale recruitment of the French Wars, often accompanied by ample bounties for joining, facilitated this type of stratagem. On 17 April 1795, Thomas Robinson volunteered for the Navy, and was paid a £10 advance over his substantial £30 enlistment bounty; on 9 June he went on board HMS *Cambridge*, and 2 days later he was discharged as ‘unserviceable’, due to a ‘lame arm’ which the captain strongly suspected to predate the enlistment. Not only did he walk away with the £10, but he subsequently applied to be paid the remainder, and Admiralty lawyers were forced to rule that he effectively had a ‘right’ to it, and his ‘disqualification’ was ‘not sufficient to justify withholding it’.^120^

Some seamen, however, may have also kept their conditions secret to avoid discharge or disgrace. This extended to officers: Carpenter Richard Bennet, 26 years of age in 1801, suffered from advanced phthisis; he had ‘recently entered into the Service, being unable to procure a living on shore owing to Ill Health—and willing to conceal His Indisposition he had not made it known to the Surgeon’.^121^ In 1806 Thomas Simpson, the surgeon of HMS *Arethusa*, found a man with the ‘itch’, and had to examine the whole crew before discovering five more who had not come forward.^122^ Venereal diseases were often kept hidden, generally to avoid the fine imposed by the Navy.^123^ Even after the fine was abolished, however, Dr Trotter noted that many seamen did not seek help because of ‘a degree of modesty… independent of other considerations’.^124^ In January 1803, during the brief Peace of Amiens, HMS *Ambuscade* was stationed in Sheerness; aboard it, William Mortimore had ‘been ailing a considerable time’ with genital chancroid (‘bubo chancres’), ‘but concealed his complaints on account of having his wife on board’—‘who’, the surgeon added, ‘I am inclined to believe must have infected him’. Perhaps the seaman wanted to spare his wife the humiliation, or perhaps he feared that she would be put ashore—women in naval ships were tolerated rather than officially allowed. He was marked as recovered after over a month of treatment, and we do not know what became of her.^125^ This kind of behaviour illustrates a grim reality, and was used by the authorities to justify the contention that seamen were like children in need of control, but it simultaneously shows us examples of personal autonomy, within a supposedly rigid system. The eye of the surgeon was not all-seeing, and the whole structure of naval medical treatment relied on seamen’s spontaneous cooperation, and voluntary application for his assistance. Sailors’ own constructions of what constituted an injury or disease in need of reporting could function along distinct lines from those of the naval establishment. Further (and this may help to avoid romanticising ‘agency’), seamen were at times structurally unable to communicate with their surgeons: more than ever, in its quest for ‘manpower’, the Navy had absorbed men whose language, in the broadest sense of the word, differed from its own—meaning that the institution could not keep track of their feelings, or even bodies.^126^ This situation differs substantially from one of ‘Foucauldian’ medicalised control.

Here we return to the young Patrick Ferreter, with whom we started this article—ignoring his luxated arm to help at a critical time, and thus likely making himself a permanent invalid. It is crucial to note that, when seamen had a motive for failing to report an ‘illness’, this could be personal advantage, but also, on the contrary, loyalty to the service, leading them to dismiss their pain in order to perform their duties. Authors celebrating the heroism of British Tars emphasised their patriotic resilience in the face of wounds, pain and death.^127^ ‘Nor does… courage ever forsake them’, wrote Thomas Trotter; ‘we have seen them cheering their shipmates, and answering the shouts of the enemy, under the most dreadful wounds, till, from loss of blood, they expired’.^128^ Seemingly improbable stories like that of a wounded seaman at the battle of Trafalgar, who refused assistance, patiently awaited his turn to be treated and then, smiling, sang ‘Rule Britannia’ as the surgeon amputated his arm, have attracted the interest of scholars of popular patriotism.^129^ State propaganda doubtlessly overplayed and simplified the ‘heroic’ element: some of these resounding accounts may leave us sceptical, blurring into nationalist trope; even ‘heroism’ itself can have several complex causes or aims. This is why a plainer, more credible story like that of Patrick Ferreter is of particular value: an untrained, non-English-speaking ‘landman’ sucked into the manpower-hungry Navy of the 1810s, and a victim of the war, he is also a direct example of how social and cultural disorientation could be mixed with sense of duty, in a unique, individual blend.

## Conclusion

Naval institutionalisation undoubtedly subjected seamen to high levels of control. However, despite their condition as the state’s ‘tools’, and the strictures of military discipline, this article has argued that in fact late eighteenth-century naval seamen had significant leeway to exert medical agency—and must be treated as central protagonists of naval medical history. Healthy and effective sailors were fundamental to the prolonged war effort: this in itself gave them bargaining power, both collectively and as individuals. Medical autonomy fit into a broader tapestry of negotiation patterns, some derived from earlier paternalistic frameworks, others conditioned by the new power dynamics and the revolutionary threat, as well as the shared knowledge, experiences and opportunities stemming from ever-increasing global deployment. Of course, life in the Navy carried high risks of disease and injury. Such risks, however, combined with the oppressing conditions of extended and (for many) forced service, often produced a very particular mindset—a conviction of not having much to lose—and a familiarity with pain and discomfort. This could lead the men to seize control of their own bodies, and use them against the very system that had first instrumentalised them. The standardised and depersonalised forms of treatment which were enhanced by large-scale mobilisation flattened the patient as an individual. Yet they could also offer him scope to wriggle out of and around the system: the unpredictability of individuals’ behaviour is key to short-circuiting the (necessarily) superficial flattening gaze and routines of the state.

At the same time, it is clear that sailors’ challenge to medical authority was not always a default cultural attitude, nor necessarily a challenge to the values and goals of the service. Some men simply sought better ways to take care of their own health, and others even sacrificed it to do what they perceived as their duty. Therefore, if depicting them as passive recipients of medical care is reductive, it would be equally reductive to cast all their decisions as calculated ‘rebellion’ or resistance. Whatever the reason—gain or desperation, shame or fear, shyness, psychological suffering, naivety or differing constructions of disease, culturally assimilated ideals of heroism and manliness, or something else altogether—seamen often behaved in ways that escaped the institutional framework and rules, using their own body as a tool, or as they pleased.

## Funding

Some revisions to this article were conducted during a Scouloudi Fellowship at the Institute of Historical Research, London, an International Fellowship at the Deutsches Schifffahrtsmuseum, Bremerhaven, and a W. M. Keck Foundation Fellowship at the Huntington Library, San Marino.

## Footnotes

1
*The London Gazette* 16750, 6 July 1813, 1329–30.

2 The National Archives, Kew [henceforth TNA], ADM 101/120/3A, Medical and Surgical Journal of HMS *Shannon* by Alexander Jack for 30 July 1812 to 29 July 1813, f. 21.

3
*Ibid*.

4
*Ibid*.

5 N. D. Jewson, ‘The Disappearance of the Sick-Man from Medical Cosmology, 1770-1870’, *International Journal of Epidemiology*, 2009, 38, 622–33.

6 Mary Fissell, *Patients, Power, and the Poor in Eighteenth-Century Bristol* (Cambridge: Cambridge University Press, 1991).

7 Christopher Lawrence, ‘Disciplining Disease: Scurvy, the Navy, and Imperial Expansion, 1750-1825’, in David Philip Miller and Peter Hanns Reill, eds, *Visions of Empire: Voyages, Botany, and Representations of Nature* (Cambridge: Cambridge University Press, 1996), 80–106, esp. 82–83, 95.

8 Peter Mathias, ‘Swords and Ploughshares: The Armed Forces, Medicine and Public Health in the Late Eighteenth Century’, in J. M. Winter, ed., *War and Economic Development: Essays in Memory of David Joslin* (Cambridge: Cambridge University Press, 1975), 73–90; Geoffrey L. Hudson, ‘Introduction: British Military and Naval Medicine, 1600-1830’, in Geoffrey L. Hudson, ed., *British Military and Naval Medicine, 1600-1830* (Amsterdam and New York: Rodopi, 2007), 7–22, 9, 12–13; Philip R. Mills, ‘Privates on Parade: Soldiers, Medicine and the Treatment of Inguinal Hernias in Georgian England’, in Hudson, *British Military and Naval Medicine*, 149–82, 151; Margarette Lincoln, *Representing the Royal Navy: British Sea Power, 1750-1815* (Aldershot and Burlington: Ashgate, 2002), 170–73; James Watt, ‘Naval and Civilian Influences on Eighteenth- and Nineteenth-Century Medical Practice’, *The Mariner’s Mirror*, 2011, 97, 148–66, 156–57, 165; Mark Harrison, ‘The Sea as a Knowledge Development Factor in the Medical Field’, in Christian Bouchet and Gérard Le Bouëdec, eds, *The Sea in History – The Early Modern World* (Woodbridge: Boydell & Brewer, 2017), 732–42. For the earlier roots of this development see: Harold J. Cook, ‘Practical Medicine and the British Armed Forces after the “Glorious Revolution”’, *Medical History*, 1990, 34, 1–26.

9 William J. Ashworth, ‘“System of Terror”: Samuel Bentham, Accountability and Dockyard Reform during the Napoleonic Wars’, *Social History*, 1998, 23, 63–79; N. A. M. Rodger, *The Command of the Ocean: A Naval History of Britain, 1649-1815* (London: Allen Lane, 2004), 451–53, 464–65, 475–80.

10 Michel Foucault, *Power/Knowledge: Selected Interviews and Other Writings 1972-1977*, Colin Gordon (ed. and trans) (Harlow: Longman, 1980), 169–77; Lincoln, *Representing*, 172, 176–78; Geoffrey L. Hudson, ‘Internal Influences in the Making of the English Military Hospital: The Early-Eighteenth-Century Greenwich’, in Hudson, *British Military and Naval Medicine*, 253–72; Lawrence, ‘Disciplining Disease’.

11 Lincoln, *Representing*, 176.

12 N. A. M. Rodger, *The Wooden World: An Anatomy of the Georgian Navy* (London: William Collins, 1986), 118; Helen Watt and Anne Hawkins, eds, *Letters of Seamen in the Wars with France 1793-1815* (Woodbridge: The Boydell Press, 2016), 34–35, 59, 479–80.

13 See, for example, most recently: Niklas Frykman, *The Bloody Flag: Mutiny in the Age of Atlantic Revolution* (Oakland: University of California Press, 2020); Callum Easton, ‘Counter-Theatre during the 1797 Fleet Mutinies’, *International Review of Social History*, 2019, 64, 389–414; Elin Jones, ‘Space, Sound and Sedition on the Royal Naval Ship, 1756-1815’, *Journal of Historical Geography*, 2020, 70, 65–73.

14 Watt, ‘Naval and Civilian Influences’, 148–66; Patricia Kathleen Crimmin, ‘British Naval Health, 1700-1800: Improvement over Time?’, in Hudson, *British Military and Naval Medicine*, 183–200; Hudson, ‘Introduction’, 18; J. D. Alsop, ‘Warfare and the Creation of British Imperial Medicine, 1600-1800’, in Hudson, *British Military and Naval Medicine*, 23–50.

15 Roy Porter, ‘The Patient’s View: Doing Medical History from Below’, *Theory and Society*, 1985, 14, 175–98; Roy Porter, ‘Introduction’, in Roy Porter, ed., *Patients and Practitioners: Lay Perceptions of Medicine in Pre-Industrial Society* (Cambridge: Cambridge University Press, 1985), 1–22; Hudson, ‘Internal Influences’, 264–66; Hudson, ‘Introduction’, 12, 18. For the traditional approach see, for example, Christopher Lloyd and Jack L. S. Coulter, *Medicine and the Navy 1200-1900: Volume III – 1714–**1815* (Edinburgh and London: E. & S. Livingstone, 1961).

16 Porter, ‘Introduction’, 2–3, 10–16.

17 Mark S. R. Jenner and Patrick Wallis, ‘The Medical Marketplace’, in Mark S. R. Jenner and Patrick Wallis, eds, *Medicine and the Market in England and Its Colonies*, c.*1450 –* c.*1850* (Basingstoke and New York: Palgrave Macmillan, 2007), 1–23; Fissell, *Patients*, 37–73; Susan C. Lawrence, *Charitable Knowledge: Hospital Pupils and Practitioners in Eighteenth-Century London* (Cambridge: Cambridge University Press, 1996), 6–7, 16–17, 336.

18 See, for example, Jerome S. Handler, ‘Slave Medicine and Obeah in Barbados, circa 1650 to 1834’, *NWIG: New West Indian Guide / Nieuwe West-Indische Gids*, 2000, 74, 57–90; Juanita De Barros, ‘“Setting Things Right”: Medicine and Magic in British Guiana, 1803-38’, *Slavery & Abolition*, 2004, 25, 28–50.

19 Rodger, *Command*, 638–39.

20 TNA, ADM 8/38, List Book, 1763; ADM 8/100, List Book, 1812–13.

21 The average displacement of line-of-battle ships was 1,960t in 1760, 2,180t in 1780, and 2,710t in 1810; for cruisers, the change was even sharper, from 810t in 1760 to 870t in 1780 and 1,220t in 1810: Jan Glete, *Navies and Nations: Warships, Navies and State Building in Europe and America, 1500-1860*, 2 vols (Stockholm: Almqvist & Wiksell International, 1993), II, 402–5.

22 Walter Johnson, ‘On Agency’, *Journal of Social History*, 2003, 37, 113–24.

23 Rodger, *Wooden World*, 205–51.

24 Catherine Beck, ‘Patronage and Insanity: Tolerance, Reputation and Mental Disorder in the British Navy 1740-1820’, *Historical Research*, 2021, 94, 73−95. See also: Roland Pietsch, ‘Hearts of Oak and Jolly Tars? Heroism and Insanity in the Georgian Navy’, *Journal for Maritime Research*, 2013, 15, 69–82.

25 See, for example, Jesse Lemisch, ‘Jack Tar in the Streets: Merchant Seamen in the Politics of Revolutionary America’, *The William and Mary Quarterly*, 1968, 25, 371–407, esp. 381–82.

26 Rodger, *Wooden World*, 86–87, 116–44; J. Ross Dancy, *The Myth of the Press Gang: Volunteers, Impressment and the Naval Manpower Problem in the Late Eighteenth Century* (Woodbridge: The Boydell Press, 2015), esp. 94–105, 187–88.

27 William Turnbull, *The Naval Surgeon* (London: Richard Phillips, 1806), vii–xiii; Lincoln, *Representing*, 162, 167; Lloyd and Coulter, *Medicine and the Navy*, 19–20.

28 Lloyd and Coulter, *Medicine and the Navy*, 10–18.

29 TNA, ADM 101, Admiralty and Predecessors, Medical Journals.

30 Watt, ‘Naval and Civilian Influences’, 156.

31 M. John Cardwell, ‘Royal Navy Surgeons, 1793-1815: A Collective Biography’, in David Boyd Haycock and Sally Archer, eds, *Health and Medicine at Sea, 1700-1900* (Woodbridge: The Boydell Press, 2009), 38–62.

32 Erica Charters, *Disease, War, and the Imperial State: The Welfare of the British Armed Forces during the Seven Years’ War* (Chicago and London: The University of Chicago Press, 2014).

33 Thomas Trotter, *Medicina Nautica: An Essay on the Diseases of Seamen*, 3 vols, 2nd edn (London: Longman, Hurst, Rees, and Orme, 1804), I, 460.

34 Alsop, ‘Warfare’, 31–33; Crimmin, ‘British Naval Health’, 187–89, 195; Mills, ‘Privates’, 162–66, 170.

35 Todd L. Savitt, ‘The Use of Blacks for Medical Experimentation and Demonstration in the Old South’, *The Journal of Southern History*, 1982, 48, 331–48.

36 Crimmin, ‘British Naval Health’, 188. The most famous experiment is described in James Lind, *A Treatise of the Scurvy* (Edinburgh: Sands, Murray, and Cochran, 1753), 87–88, 191–96.

37 Lawrence, *Charitable Knowledge*, 297–301, 323–24.

38 Susan E. Lederer, ‘The Ethics of Experimenting on Human Subjects’, in Robert B. Baker and Laurence B. McCullough, eds, *The Cambridge World History of Medical Ethics* (Cambridge: Cambridge University Press, 2008), 558–65; Ulrich Tröhler, ‘The Historical Development of International Codes of Ethics for Human Subjects Research’, in Baker and McCullough, eds, *History of Medical Ethics*, 566–75.

39 Tröhler, ‘Historical Development’, 567.

40 James Carmichael Smyth, *An Account of the Experiment Made at the Desire of the Lords Commissioners of the Admiralty, on Board the Union Hospital Ship, to Determine the Effect of the Nitrous Acid in Destroying Contagion, and the Safety with Which It May Be Employed* (London: J. Johnson, 1796), 12, 30–31.

41 Alsop, ‘Warfare’, 28–30, 35–38; Lincoln, *Representing*, 168–69; Crimmin, ‘British Naval Health’. For soldiers, see Mills, ‘Privates’, 162–63.

42 Foucault, *Power/Knowledge*, 169–72; Hudson, ‘Internal Influences’, 258.

43 Erica M. Charters, ‘“The Intention Is Certain Noble”: The Western Squadron, Medical Trials, and the Sick and Hurt Board during the Seven Years War (1756-63)’, in Haycock and Archer, eds, *Health and Medicine*, 30–3; Charters, *Disease*, 132–40.

44 See, for example, Gilbert Blane, *Observations on the Diseases of Seamen*, 2nd edn (London: Joseph Cooper, 1789), 211–367; Turnbull, *Naval Surgeon*, 1–53.

45 Thomas Reynolds, ‘Proposals for a Regulation of the Oeconomy of the Sick on Board His Majesty’s Ships of War at Sea’, in *The Gentleman’s Magazine*, 1758, 28, 61; I found this article through a mention in Lincoln, *Representing*, 165.

46 Lind, *Treatise*, 188–89; Anon, ‘A Method for Preserving the Health of Seamen in Long Cruizes and Voyages’, in *The Gentleman’s Magazine*, 1747, 17, 468; Blane, *Observations*, 116.

47 Reynolds, ‘Proposals’, 106; Blane, *Observations*, 147, 151; Lincoln, *Representing*, 165, 172–73.

48 On this shift see Lawrence, ‘Disciplining Disease’.

49 Blane, *Observations*, 53–54, 78–80, 99, 102, 108, 114–15, 216, 222–23.

50
*A Voyage to St. Petersburg in 1814, with Remarks on the Imperial Russian Navy. By a Surgeon in the British Navy* (London: Sir Richard Phillips & Co., 1822), 13, 20–21.

51
*Ibid*., 13.

52 Turnbull, *Naval Surgeon*, xxv–vi; Alsop, ‘Warfare’, 37–9; Lincoln, *Representing*, 174–75, 179.

53 Reynolds, ‘Proposals’, 64, 158.

54 Caird Library, National Maritime Museum, Greenwich (henceforth NMM), ADM/E/46, Sick and Hurt Board, In-Letters and Orders, 1797–8, T. Trotter to Evan Nepean, 30 August 1797.

55 Jane Bowden-Dan, ‘Diet, Dirt and Discipline: Medical Developments in Nelson’s Navy. Dr John Snipe’s Contribution’, *The Mariner’s Mirror*, 2004, 90, 260–72, 264, 268–69; Lloyd and Coulter, *Medicine and the Navy*, 161–64. For some recent contributions on the 1797 fleet mutinies and sailors’ agency see: Ann Veronica Coats and Philip MacDougall, eds, *The Naval Mutinies of 1797: Unity and Perseverance* (Woodbridge: The Boydell Press, 2011); Easton, ‘Counter-Theatre’.

56 Hudson, ‘Internal Influences’, 264–67.

57 TNA, ADM 101/95/1, Medical and surgical journal of His Majesty’s Brig *Contest* for 30 September 1807 to 1 October 1808 by Thomas T. Crew, Acting Assistant Surgeon, fos. 6–8.

58 Kevin White (ed.) and J. T. Alridge, *The Early Sociology of Health and Illness – Volume 6: Hygiene, Diseases and Morality of Occupations* (London: Routledge, 2001), 346–49.

59 TNA, ADM 101/107/1, Medical journal of His Majesty's Ship *London* for 17 February to 25 August 1800 by James Little, Surgeon, f. 31.

60 TNA, ADM 101/121/3B, Medical journal of His Majesty's Ship *Swiftsure* for 8 July 1798 to 9 July 1799 by James Dalziel, Surgeon, f. 12; ADM 101/93/2D, Journal of HMS *Captain* by James Farquhar, Surgeon, between 3 September 1798 and 26 May 1799, f. 3. On modern legal and moral controversies concerning mandatory vaccination in the armed forces see, for example, Randall D. Katz, ‘Friendly Fire: The Mandatory Military Anthrax Vaccination Program’, *Duke Law Journal*, 2001, 50, 1835–65.

61 Crimmin, ‘British Naval Health’, 195.

62 Jenner and Wallis, *Medicine and the Market.*

63 Sara Caputo, ‘Towards a Transnational History of the Eighteenth-Century British Navy’, *Annales Historiques de la Révolution Française*, 2019, 397, 13–32.

64 On sailors becoming victims of fraudsters ashore see, for example, Pietsch, ‘Hearts of Oak’, 78–80.

65 Turnbull, *Naval Surgeon*, 82. On the history of the fine see: Lloyd and Coulter, *Medicine and the Navy*, 357–58.

66 Trotter, *Medicina Nautica*, I, 460, 462. On the profitable business of venereal treatments see: W. F. Bynum, ‘Treating the Wages of Sin: Venereal Disease and Specialism in Eighteenth-Century Britain’, in W. F. Bynum and Roy Porter, eds, *Medical Fringe and Medical Orthodoxy, 1750-1850* (London: Croom Helm, 1987), 5–28.

67 TNA, ADM 101/115/3B, Medical journal of HMS *Princess Royal* for 7 February to 17 April 1802 Ben Lara, Surgeon, fos. 5–6. I am very grateful to one of the anonymous referees for directing me to this specific journal.

68 John D. Byrn, *Crime and Punishment in the Royal Navy: Discipline on the Leeward Islands Station, 1784-1812* (Aldershot: Scolar Press, 1989), 165; Nick Slope, ‘Discipline, Desertion and Death: HMS *Trent*, 1796-1803’, in Coats and MacDougall, *Naval Mutinies*, 226–42, 231–32. For an earlier period see: Rodger, *Wooden World*, 199–204; Rodger, *Command*, 318.

69 TNA, ADM 101/112/5, Medical journal of His Majesty's Prison Hospital ship *Le Pegaze* [*Le Pegase*] from the 25 January 1804 to 14 January 1805 by [William Bickley Smith?], Surgeon, f. 4.

70 Blane, *Observations*, 50–51; TNA, ADM 98/24, Sick and Hurt Board to the Admiralty, 1806–8, fos. 77–78, 109–10, 6 July, 18 August 1807.

71
*Regulations and Instructions Relating to His Majesty’s Service at Sea*, 13th edn (London, 1790), 200, xi.4.

72 Watt and Hawkins, *Letters of Seamen*, 112–13.

73
*Ibid*., 148.

74
*Ibid*., 112–13.

75 Wendy D. Churchill, ‘Efficient, Efficacious and Humane Responses to Non-European Bodies in British Military Medicine, 1780-1815’, *The Journal of Imperial and Commonwealth History*, 2012, 40, 137–158, 142–45.

76 On doctors’ views see *Ibid*., 145–46.

77 Lloyd and Coulter, *Medicine and the Navy*, 191, 252–54.

78 TNA, ADM 102/282, Hospital Muster Books – Haslar, 1798; ADM 102/286, Hospital Muster Books – Haslar, 1801; ADM 102/297, Hospital Muster Books – Haslar, 1813.

79 TNA, ADM 102/612, Hospital Muster Books – Plymouth, 1801, January, fos. 9, 35.

80 Guenter B. Risse, ‘Britannia Rules the Seas: The Health of Seamen, Edinburgh, 1791-1800’, *The Journal of the History of Medicine and Allied Sciences*, 1988, 43, 426–46, 430–32.

81 TNA, ADM 98/18, Sick and Hurt Board to the Admiralty, 1796–8, fos. 78–79, 18 November 1796; Jonathan Coad, ‘To Serve the Fleet in Distant Waters. Buildings of the Georgian Royal Navy’s Overseas Bases’, *Transactions of the Naval Dockyards Society*, 2009, 5, 51–63, 61.

82 NMM, ADM/E/46, Evan Nepean, 15 April 1797, attachment from Captain Yeo.

83
*Ibid*.

84 NMM, ADM/E/46, R. R. Burges et al. to Admiral Onslow, enclosure to letter by William Marsden, 15 May 1797.

85 See, for example, Roger Cooter, ‘Malingering in Modernity: Psychological Scripts and Adversarial Encounters during the First World War’, in Roger Cooter, Mark Harrison and Steve Sturdy, eds, *War, Medicine and Modernity* (Stroud: Sutton Publishing, 1998), 125–48; Donald Lee Anderson and Godfrey Tryggve Anderson, ‘Nostalgia and Malingering in the Military during the Civil War’, *Perspectives in Biology and Medicine*, 1984, 28, 156–66, 161–65; R. Gregory Lande and Lisa Banks Williams, ‘Prevalence and Characteristics of Military Malingering’, *Military Medicine*, 2013, 178, 50–54; R. Gregory Lande, *Madness, Malingering, and Malfeasance: The Transformation of Psychiatry and the Law in the Civil War Era* (Washington DC: Brassey’s, Inc., 2003), esp. 131–56, 166–67.

86 Richard A. A. Kanaan and Simon C. Wessely, ‘The Origins of Factitious Disorder’, *History of the Human Sciences*, 2010, 23, 68–85; Cooter, ‘Malingering’; Sebastian Schnellbacher and Heather O’Mara, ‘Identifying and Managing Malingering and Factitious Disorder in the Military’, *Current Psychiatry Reports*, 2016, 18, 1–7, 1.

87 Rodger, *Command*, 497–500.

88 Schnellbacher and O’Mara, ‘Identifying and Managing Malingering’, 2.

89 On the evolution of medical ‘sleuthing’ techniques in this sense see: Cooter, ‘Malingering’, 127–29.

90 Fissell, *Patients*, 148–55.

91 TNA, ADM 101/97/2, Medical journal of HMS *Dreadnought* for 15 March to 8 October 1803 by [Thomas] Carruthers, Surgeon, fos. 47–49.

92
*Ibid*., f. 20.

93 NMM, ADM/E/46, Evan Nepean, 15 April 1797, attachment.

94
*A Narrative of Joshua Davis, an American Citizen, Who Was Pressed and Served on Board Six Ships of the British Navy* (Boston: B. True, 1811), 50–54.

95
*Narrative of the Travels and Voyages of Davis Bill* (Brattleborough: William Fessenden, [1810]), 99–100.

96 TNA, ADM 101/93/2C, Journal of HMS *Captain* for 27 May 1798 to 2 September 1798 by James Farquhar, Surgeon, f. 6.

97 Rodger, *Command*, 497.

98 See, for example, TNA, ADM 101/125/3, Medical and surgical journal of His Majesty’s Ship *Ville de Paris* for 25 March 1813 to 24 March 1814 by William Warner, Surgeon, f. 16; ADM 101/107/2A, Medical journal of His Majesty's Ship the *London* from 24 July 1800 to 25 April 1801 by James Little, Surgeon, f. 22.

99 TNA, ADM 101/106/4, Medical and surgical journal of His Majesty's *Lion* for 29 January 1813 to [29th] January 1814 by John Tweedy Todd, Surgeon, fos. 26, 35.

100 TNA, ADM 101/86/1, Thomas Simpson, Journal of His Majesty’s Ship *Arethusa*, 14 May 1805 to 14 June 1806 [f. 11].

101
*Ibid.*

102 TNA, ADM 101/84/6A, Medical journal HMS *Ambuscade* for 26 August 1800 to 27 August 1801 by Thomas Hendry, f. 19.

103 TNA, ADM 102/612, March, f. 17.

104 TNA, ADM 101/111/5, Medical journal of HMS *Overyssel*, 1797–98, f. 34.

105 Frykman, *Bloody Flag*.

106 NMM, ADM/E/46, Evan Nepean, 16 May, 27 May, 8 June 1797.

107 On this dilemma see also Cooter, ‘Malingering’, 131–32.

108 Pietsch, ‘Hearts of Oak’, 71–74.

109
*Recollections of James Anthony Gardner Commander R.N. (1775-1814)*, R. Vesey Hamilton and John Knox Laughton, eds (London: Navy Records Society, 1906), 31–32.

110 [Charles McPherson], *Life on Board a Man-of-War* (Glasgow: Blackie, Fullarton, & Co., 1829), 41–44.

111 For some present-day examples see: Arnon D. Cohen and Daniel A. Vardy, ‘Dermatitis Artefacta in Soldiers’, *Military Medicine*, 2006, 171, 497–99; Russell Pokroy and Arie Marcovich, ‘Self-Inflicted (Factitious) Conjunctivitis’, *Ophthalmology*, 2003, 110, 790–95.

112 Dancy, *Myth*; Nicholas Rogers, ‘British Impressment and Its Discontents’, *The International Journal of Maritime History*, 2018, 30, 52–73.

113 Trotter, *Medicina Nautica*, 40–41.

114
*Ibid.*

115 Hector Gavin, *On Feigned and Factitious Diseases, Chiefly of Soldiers and Seamen, on the Means Used to Simulate or Produce Them, and on the Best Modes of Discovering Impostors* (London: John Churchill, 1843).

116 TNA, ADM 12/129, Admiralty Digest 1807 – Part 3, 79.17, summary of letters from Admiral Douglas, 29 March, 1 April, 6 April 1807.

117 John D. Byrn (ed.), *Naval Courts Martial, 1793-1815* (Farnham and Burlington: Routledge, 2009), 288–93.

118 TNA, ADM 1/1066, Letters from Commanders-in-Chief, Portsmouth, 1804, nn. 153–300, A281, George Montagu to William Marsden, 4 March 1804; ADM 12/67, Admiralty Digest 1795 – Part 2, 61, summary of letter from Admiral Peter Parker, 20 December 1795; ADM 1/2134, Letters from Captains, Surnames M: 1797, nn. 201–363, 329, Sylvester Moriarty to Evan Nepean, 10 December 1797.

119 See, for example, Lande, *Madness*, 138.

120 TNA, ADM 7/302, Law Officers’ Opinions, 1793–5, n. 30, Case of Thomas Robinson, 16 June 1796.

121 TNA, ADM 101/84/6A, f. 16.

122 TNA, ADM 101/86/1, f. 6].

123 Turnbull, *Naval Surgeon*, 82–83.

124 Trotter, *Medicina Nautica*, I, 459.

125 TNA, ADM 101/84/6D, Medical journal HMS *Ambuscade* for 28 September 1802 to 15 March 1803 by J Anderson, fos. 11–16.

126 See, for example, Churchill, ‘Efficient, Efficacious and Humane Responses’, 147–48.

127 Lincoln, *Representing*, 174, 177; Pietsch, ‘Hearts of Oak’, 74, 76–77.

128 Trotter, *Medicina Nautica*, 37.

129 Linda Colley, ‘Britishness and Otherness: An Argument’, *Journal of British Studies*, 1992, 31, 309–29, 32–33.

## Notes

Sara Caputo completed her PhD at the University of Cambridge in 2019, and is now Lumley Junior Research Fellow at Magdalene College, University of Cambridge. She works on the transnational social and cultural history of eighteenth-century maritime communities.

